# Malaria

**Published:** 1894-03-24

**Authors:** 


### Medical Progress.

### MALARIA.

*Plasmodium, Effect of Drugs on.*—The ready demonstration of the hæmatozoa has become a matter of practical value,[1] often as necessary as that of the tubercle bacillus. Half the mortality in the tropics is due to their influence, and even here, in all seaports, it is at times impossible to tell what acute disease we are dealing with unless we can decide whether they are present or not.

They have not been found in the blood apart from malaria, and the injection of blood containing them has produced malaria in healthy persons. The tertian, quartan, and cyclical types are now distinguished by some authorities. Manson[2] directs that a very small drop of blood should be obtained by pricking the finger. Wipe off the first that comes, and touch the second with the centre of a thin cover glass. This should be dropped on a slip, so that as thin a layer of blood as possible with the corpuscles lying separate and flat be obtained. Examine at once, or seal with vaseline, and use two powers of 300 and 600 diameters. Minute specks of dark pigment in a transparent mass, circular, crescent, sausage-shaped, or like rosettes, and occasionally with flagellæ, may be found, one or two in a field. They may be within or outside corpuscles. If desired, they can be stained with methylene blue. Osler[3] only missed them in eight out of seventy cases, and five of these had been under quinine.

Rosin[4] found that the movements of these plasmodia are not stopped if quinine (1 in 5,000) be run in under the cover glass, but methylene blue (1 in 20,000) killed them at once. The latter acts in the blood as a "leuco" compound, and Matienzo[5] and others recommend it in certain cases as an efficient substitute for quinine, especially in those of a mild type. Whether the various forms are distinct organisms or phases in the life history of one germ is not quite certain. However, some few cases are refractory to quinine, some to phenocoll, some chronic cases yield to arsenic, and some only improve by change of residence. Ch. Forbes recommends eucalyptus, and asserts the value of opium as a prophylactic. This appears to be the popular belief in India, and Surgeon-Major Laurie,[6] giving charts of two cases, delares that it actually reduces the fever and vomiting as well as the pain in the disease itself. Europeans usually employ quinine, which is more certain, and can be bought at all post offices at a farthing for five grains of the Government alkaloids. There is much conflict of opinion as to the prophylactic power of opium, and some of its supporters ascribe its value to the large amount of narcotine contained in the Indian drug.[7] Whether it be a true prophylactic or no, great relief from the pain and bowel complaints and increased ability for work is obtained in bad malarial districts by its use. The whole question is being argued before the Opium Commission[8] at the present time. Moreover, quinine is not without its dangers in certain types of malaria, and produces serious results. Binz[9] holds that quinine acts on the protozoa directly as a poison more than it does on the cells of the human body. Where it fails when given by the mouth intravenous injections may succeed. Councilman thinks that it has no effect on the crescentic organisms which occur in some of the more chronic forms of the disease.

Cerna, in an elaborate paper describes his results with PHENOCOLL.[10] He treated 28 cases with it alone, 21 of which were successful, and three of the others yielded to quinine. The doses were one, two, and even three grammes daily for four or five days. Even where it failed it reduced the temperature. P. Pucci[11] and others confirm his results and regard it as a valuable substitute for quinine when the latter produces ictero-hæmaturic fever.

Cervello treated 15 cases by it and Banetti 42 with success, and Ferreira writes strongly on its value even in the case of infants. T. S. Short[12] records a case of malarial paroxysms with high temperatures, rigors, &c., occurring in the fourth week of typhoid. The two diseases ran an independent course.

*Actinomycosis.*—The marked remedial action of iodide of potash both in cattle and men is the subject of papers by Ransom and Valerio.[13] In daily doses of one or two drachms it appears to effect a complete cure. Dr. Kanthack, in showing a specimen of the pyæmic form remarks that clubs are frequently absent in undoubted cases of the disease.

*Glanders*[14] in London has been reduced by two-thirds under the new rules. The bacillus mallei may survive on dry manure for three months and produce the disease in man,[15] which at first may be indistinguishable from rheumatic fever. Hell and Tœpper have stopped outbreaks by injecting the serum of convalescent animals.

Cases of foot and mouth disease in man[16] are reported from Berlin. The tongue swells very much and may become gangrenous, blisters occur on the nose, mouth, prepuce, with a prolonged irregular fever and albuminuria.[17]

*Epidemic Meningitis.*[18]— The eye symptoms are a well marked feature; not only conjunctivitis and keratitis, but also optic neuritis, engorgement and tortuosity of the retinal veins are common, the right eye being affected most frequently. Klemperer[19] points out the value of labial herpes in diagnosing epidemic from the tubercular form. This herpes is probably not of the nature of zona, and is caused by a localization of the microbe of the general disease. It is common in affections where micrococci are found, *e.g.*, pneumonia and endocarditis, but very rare where special bacilli exist, as in typhoid or tubercle. Cocci are often found in the vesicles, like those of the systemic disease. The Cologne epidemic which began in 1885 produced 194 cases. The germ is held by Leichtenstern[20] to be a specialised form derived from Fränkel's diplococcus, which once developed maintains its peculiarities, but has little vital capacity and soon dies out. In contrast with these results, diseases produced by streptococci have been shown by Goldscheider[21] not to depend on varieties of the germ but on differences in its habitat, especially on the presence of necrosed tissue.

1 Glasgow Med., Jan., 1894. 2 Lancet, Feb. 6. 3 B. M. J., Feb. 10. 4 B. M. J., Dec. 30, 1893. 5 Am. J. Med. S., Nov., 1893. 6 B. M. J., Jan. 27, 1894. 7 B. M. J., Dec., Jan., Feb., 1894. 8 Ch. Miss. Quar., Jan. 1894. 9 Centrall f. Med. Wiss, 2, 1894. 10 Ther. Gaz., Dec. 1893. 11 B. M. J., Dec., 1893; and Les Nouv. Remedes, Dec., 1893. 12 Birm. Med. Rev., Jan., 1894. 13 B. M. J., Jan. 6; Ther. Gaz., Jan., 1894. 14 B. M. J., Jan. 27, 1894. 15 B. M. J., Feb. 10. 16 Practit, Nov., 1893. 17 Am. J., Med. S., Dec. 1893. 18 Am. J., Med. S., Feb. 1894. 19 Am. J., Med. S., Feb., 1894. 20 Prov. Med. J.. Feb. 1894. 21 Centrall f. Klin. Med. 33, 1893.

